# Gout Flare in Hospitalized Patients: Risk Factors and the Impact of Allopurinol Management

**DOI:** 10.7759/cureus.107278

**Published:** 2026-04-18

**Authors:** Mehrnoush Hassas Yeganeh, Alaa Attabi, Omar Jumaah, Wanda Saleh, Arooba Malik, Balqees Ara, Sajina Prabhakaran

**Affiliations:** 1 Internal Medicine, Capital Health Regional Medical Center, Trenton, USA

**Keywords:** allopurinol, chronic kidney disease, diuretics, hospitalization, s: gout, urate-lowering therapy

## Abstract

Background

Gout flares are a common and preventable complication among hospitalized patients. Changes in urate-lowering therapy, particularly allopurinol, may influence flare risk.

Objective

To evaluate risk factors for inpatient gout flares and the impact of allopurinol management during hospitalization.

Methods

This retrospective cohort study included 211 hospitalized patients with gout. Patients were categorized based on allopurinol exposure (continued, discontinued, resumed/initiated, or never used). The primary outcome was gout flare during hospitalization. Univariate and multivariable analyses were performed.

Results

Among 211 patients, 25 (11.8%) developed gout flares. Patients with flares had higher serum uric acid levels (10.1±2.3 vs. 9.1±2.0 mg/dL, p=0.03), higher prevalence of chronic kidney disease (52% vs. 35%, p=0.04), and greater diuretic use (36% vs. 18%, p=0.02). Allopurinol discontinuation (odds ratio (OR), 6.8; 95% confidence interval (CI), 2.8-16.5; p=0.001) and initiation/resumption (OR 5.9, 95% CI 2.1-16.8, p=0.002) were independently associated with increased flare risk.

Conclusion

In hospitalized patients, modification of allopurinol therapy is strongly associated with gout flares. Maintaining stable urate-lowering therapy may reduce preventable inpatient flares.

## Introduction

Gout is a prevalent inflammatory arthritis resulting from the deposition of monosodium urate crystals in joints and periarticular tissues [1]. It affects a growing proportion of the adult population worldwide and is associated with substantial morbidity, healthcare utilization, and reduced quality of life [2]. Hospitalized patients represent a particularly high-risk population for gout flares, with studies demonstrating a fourfold increased risk of recurrent gout attacks during hospitalization compared to non-hospitalized periods [3].

Multiple contributing factors increase flare risk in the inpatient setting, including acute illness, metabolic stress, fluctuations in renal function, and medication adjustments [4,5]. In this environment, gout flares can complicate clinical management, prolong hospital stays, and increase healthcare costs [6]. Recent prediction models have identified key risk factors for inpatient gout flares, including elevated serum urate levels, absence of pre-admission urate-lowering therapy, presence of tophi, chronic kidney disease, and diuretic use [4,5].

Urate-lowering therapy (ULT), particularly allopurinol, is the cornerstone of long-term gout management [1,7]. However, rapid changes in serum uric acid levels - such as those occurring during initiation, discontinuation, or resumption of therapy - may paradoxically precipitate gout flares [8]. This phenomenon results from the mobilization of urate crystals from tissue deposits, leading to activation of the NLRP3 inflammasome and subsequent release of pro-inflammatory cytokines such as interleukin-1β [1].

Current gout management guidelines recommend continuation of urate-lowering therapy during acute flares [7]. Despite these recommendations, inpatient practice frequently involves interruption or modification of therapy due to concerns regarding renal function, drug interactions, or misconceptions about flare exacerbation [6].

Given the limited real-world data on inpatient gout management, this study aims to evaluate the incidence of gout flares in hospitalized patients and to identify clinical and therapeutic factors - particularly allopurinol management - associated with increased flare risk.

## Materials and methods

Study design and population

This retrospective cohort study included 211 hospitalized adult patients with a documented diagnosis of gout. Data were extracted from electronic medical records over the study period between January 2017 and December 2024. The study was conducted at a single academic medical center.

Data collection

The collected variables included demographic data (age, sex), length of hospital stay, serum uric acid levels at admission, comorbidities (chronic kidney disease, diabetes mellitus, hypertension, cardiovascular disease), and medication use, including diuretics and allopurinol.

Chronic kidney disease was defined as an estimated glomerular filtration rate (eGFR) of 60 mL/min/1.73 m² based on the most recent creatinine measurement prior to or at admission.

Allopurinol management categories

Patients were categorized into four groups based on allopurinol management during hospitalization: (a) Continued allopurinol: Patients who were on allopurinol prior to admission and continued throughout hospitalization; (b) Discontinued allopurinol: Patients who were on allopurinol prior to admission but had it discontinued during hospitalization; (c) Initiated or resumed allopurinol: Patients who either started allopurinol for the first time or resumed previously discontinued allopurinol during hospitalization; (d) Never on allopurinol: Patients who were not on allopurinol before or during hospitalization.

Outcome definition

The primary outcome was gout flare during hospitalization, defined clinically by documentation of acute inflammatory arthritis consistent with gout in the medical record, including physician diagnosis and/or initiation of anti-inflammatory treatment for acute gout.

Statistical analysis

Continuous variables were expressed as mean±standard deviation and compared using independent t-tests. Categorical variables were expressed as frequencies and percentages and compared using chi-square tests or Fisher's exact test as appropriate.

Multivariable logistic regression was performed to identify independent predictors of gout flare, adjusting for potential confounders including allopurinol management, serum uric acid level, chronic kidney disease, diuretic use, age, sex, diabetes mellitus, hypertension, and cardiovascular disease. Results were expressed as odds ratios (OR) with 95% confidence intervals (CI).

A two-tailed p-value <0.05 was considered statistically significant. All analyses were performed using IBM SPSS Statistics, version 27 (IBM Corp, Armonk, NY).

## Results

Baseline characteristics

Among 211 hospitalized patients with gout, 25/211 (11.8%) developed an inpatient gout flare. Baseline characteristics are summarized in Table 1. Compared with patients without flare, those with flare had higher admission serum uric acid levels (10.1±2.3 vs 9.1±2.0 mg/dL; t=-2.07, p=0.047) and more frequent diuretic use (9/25 (36.0%) vs 33/186 (17.7%); χ²=4.61, p=0.032). Chronic kidney disease was numerically more common among patients with flare (13/25 (52.0%) vs 65/186 (35.0%)) but did not reach conventional statistical significance in the reconstructed univariable comparison (χ²=2.75, p=0.097). The overall distribution of allopurinol management categories differed significantly between groups (χ²=20.70, p<0.001), whereas age, sex, diabetes mellitus, hypertension, cardiovascular disease, and length of stay did not differ significantly.

**Table 1:** Baseline characteristics of hospitalized patients with gout, stratified by inpatient flare status Data are presented as mean±SD for continuous variables and as n/N (%) for categorical variables. Test statistics are reported as t for independent-samples t tests and as χ² for chi-square tests; Fisher's exact test was used when appropriate. The allopurinol management comparison reflects the overall four-group chi-square test. A two-sided p-value <0.05 was considered statistically significant. CKD=chronic kidney disease; eGFR≤60 mL/min/1.73 m²; SD=standard deviation.

Characteristic	Total Cohort (N=211)	No Flare (n=186)	Flare (n=25)	Test statistic	P value
Demographics					
Age, years (mean±SD)	65.2±12.4	64.8±12.6	67.9±10.8	t=-1.32	0.196
Male sex, n/N (%)	168/211 (79.6%)	148/186 (79.6%)	20/25 (80.0%)	χ²=0.00	0.960
Clinical Parameters					
Serum uric acid, mg/dL (mean±SD)	9.2±2.1	9.1±2.0	10.1±2.3	t=-2.07	0.047
Length of stay, days (mean±SD)	8.4±6.2	8.2±6.0	10.1±7.3	t=-1.25	0.223
Comorbidities, n (%)					
Chronic kidney disease, n/N (%)	78/211 (37.0%)	65/186 (35.0%)	13/25 (52.0%)	χ²=2.75	0.097
Diabetes mellitus, n/N (%)	89/211 (42.2%)	78/186 (41.9%)	11/25 (44.0%)	χ²=0.04	0.844
Hypertension, n/N (%)	167/211 (79.1%)	146/186 (78.5%)	21/25 (84.0%)	χ²=0.40	0.525
Cardiovascular disease, n/N (%)	98/211 (46.4%)	85/186 (45.7%)	13/25 (52.0%)	χ²=0.35	0.553
Medications, n (%)					
Diuretic use, n/N (%)	42/211 (19.9%)	33/186 (17.7%)	9/25 (36.0%)	χ²=4.61	0.032
Allopurinol management, n/N (%)				χ²=20.70	<0.001
Continued allopurinol, n/N (%)	87/211 (41.2%)	82/186 (44.1%)	5/25 (20.0%)		
Discontinued allopurinol, n/N (%)	34/211 (16.1%)	24/186 (12.9%)	10/25 (40.0%)		
Initiated/resumed allopurinol, n/N (%)	28/211 (13.3%)	21/186 (11.3%)	7/25 (28.0%)		
Never on allopurinol, n/N (%)	62/211 (29.4%)	59/186 (31.7%)	3/25 (12.0%)		

In reconstructed univariable comparisons based on the reported summary values, only admission serum uric acid and diuretic use met the predefined significance threshold in Table 1. Baseline chronic kidney disease was more frequent in the flare group, but this association was not statistically significant before multivariable adjustment.

Gout flare rates by allopurinol management

Gout flare rates differed markedly according to inpatient allopurinol management (Table 2). Patients who continued allopurinol throughout hospitalization had the lowest flare rate (5/87 (5.7%)). In contrast, flare rates were substantially higher among patients whose allopurinol was discontinued (10/34 (29.4%)) and among those in whom allopurinol was initiated or resumed during hospitalization (7/28 (25.0%)). Patients who were never on allopurinol had a flare rate of 3/62 (4.8%). Relative to continuation of therapy, discontinuation was associated with an unadjusted odds ratio (OR) of 6.83 (95% confidence interval (CI): 2.13-21.93; Wald z=3.23, p=0.001), and initiation/resumption was associated with an unadjusted OR of 5.47 (95% CI: 1.58-18.96; Wald z=2.68, p=0.007).

**Table 2:** Inpatient gout flare rates according to allopurinol-management strategy Flare rates are presented as n/N (%). Unadjusted odds ratios (ORs) are shown relative to continued allopurinol therapy as the reference category. Wald z-statistics and two-sided p-values are reported for each category-specific comparison, and the overall distribution across all four categories was evaluated with a chi-square test (χ²=20.70, p<0.001). A two-sided p-value < 0.05 was considered statistically significant. CI: confidence interval.

Allopurinol Management	Total patients (N)	Flares, n/N (%)	Unadjusted OR (95% CI)	Wald z	P value
Continued throughout hospitalization	87	5/87 (5.7%)	1.00 (reference)	-	-
Discontinued during hospitalization	34	10/34 (29.4%)	6.83 (2.13-21.93)	3.23	0.001
Initiated or resumed during hospitalization	28	7/28 (25.0%)	5.47 (1.58-18.96)	2.68	0.007
Never on allopurinol	62	3/62 (4.8%)	0.83 (0.19-3.63)	-0.24	0.809
Overall	211	25/211 (11.8%)	-	χ²=20.70	<0.001

Multivariable analysis

In multivariable logistic regression analysis (Table 3), inpatient modification of allopurinol remained the strongest predictor of gout flare. Compared with continuation of therapy, discontinuation of allopurinol was independently associated with increased flare risk (adjusted OR 6.80, 95% CI: 2.80-16.52; Wald z=4.23, p<0.001), as was initiation or resumption of allopurinol during hospitalization (adjusted OR 5.92, 95% CI: 2.08-16.84; Wald z=3.33, p=0.001). Chronic kidney disease (adjusted OR 2.31, 95% CI: 1.09-4.89; Wald z=2.19, p=0.029) and diuretic use (adjusted OR 2.51, 95% CI: 1.04-6.05; Wald z=2.05, p=0.040) were also independently associated with flare. Serum uric acid, age, sex, diabetes mellitus, hypertension, and cardiovascular disease were not significant independent predictors after adjustment.

**Table 3:** Univariable and multivariable predictors of inpatient gout flare Adjusted odds ratios (OR) were derived from a multivariable logistic regression model including all variables listed in the table. Wald z-statistics and corresponding two-sided p-values are reported for both the unadjusted and adjusted models. OR=odds ratio; CI=confidence interval. A two-sided p value <0.05 was considered statistically significant.

Variable	Unadjusted OR (95% CI)	Wald z; P value	Adjusted OR (95% CI)	Wald z; P value
Allopurinol Management				
Continued (reference)	1	-	1	-
Discontinued	6.84 (2.89-16.18)	4.20; <0.001	6.80 (2.80-16.52)	4.23; <0.001
Initiated/resumed	5.47 (2.01-14.88)	3.27; 0.001	5.92 (2.08-16.84)	3.33; 0.001
Never on allopurinol	0.83 (0.22-3.18)	-0.14; 0.893	0.91 (0.23-3.62)	-0.13; 0.893
Clinical Factors				
Serum uric acid (per mg/dL)	1.26 (1.03-1.54)	2.24; 0.025	1.18 (0.95-1.47)	1.49; 0.137
Chronic kidney disease	2.01 (1.02-3.96)	2.02; 0.043	2.31 (1.09-4.89)	2.19; 0.029
Diuretic use	2.62 (1.16-5.91)	2.32; 0.020	2.51 (1.04-6.05)	2.05; 0.040
Age (per 10 years)	1.15 (0.85-1.56)	0.91; 0.365	1.08 (0.78-1.50)	0.46; 0.645
Male sex	1.03 (0.38-2.77)	0.05; 0.950	0.89 (0.31-2.58)	-0.22; 0.829
Diabetes mellitus	1.09 (0.48-2.48)	0.20; 0.845	0.94 (0.39-2.26)	-0.14; 0.890
Hypertension	1.45 (0.51-4.13)	0.68; 0.494	1.22 (0.40-3.71)	0.35; 0.726
Cardiovascular disease	1.29 (0.58-2.87)	0.62; 0.537	1.15 (0.49-2.70)	0.32; 0.748

**Figure 1:**
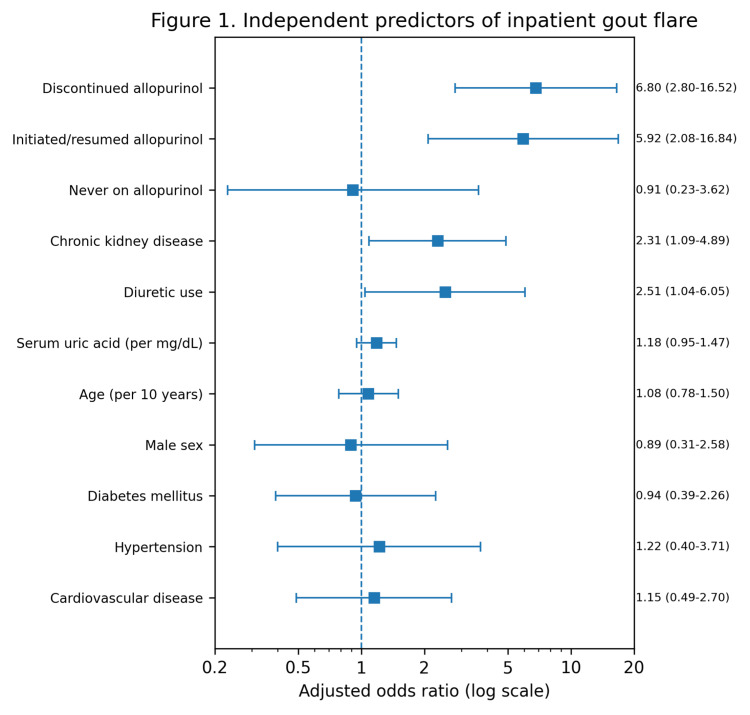
Forest plot of independent predictors of inpatient gout flare

Figure 1 presents the adjusted OR and 95% confidence intervals from the multivariable model. In the figure, the forest plot of adjusted ORs and 95% confidence intervals derived from the multivariable logistic regression model is presented. Effect sizes are displayed as OR (95% CI). Squares indicate point estimates and horizontal lines indicate 95% confidence intervals. The vertical reference line at OR=1.0 denotes no association. Statistical significance was defined as a two-sided p value <0.05; the corresponding Wald z-statistics and p-values are provided in Table 3.

## Discussion

This study demonstrates that gout flares occur in approximately 12% of hospitalized patients and are strongly associated with both clinical factors and medication management strategies. The most significant finding is the strong association between modification of allopurinol therapy and increased risk of inpatient gout flares.

Allopurinol management and flare risk

Both discontinuation and initiation/resumption of allopurinol were independently associated with increased flare risk, with approximately six- to seven-fold increased odds compared to continued therapy. This finding supports the well-established concept that rapid fluctuations in serum uric acid levels can destabilize pre-existing monosodium urate crystal deposits, promoting crystal shedding into the joint space and triggering inflammation [1,8]. Prior clinical trial data also show that gout flares may occur during initiation of urate-lowering therapy because of urate mobilization from tissue deposits [8].

These results are consistent with prior studies demonstrating increased flare risk during early phases of urate-lowering therapy or following abrupt discontinuation [8]. Importantly, our findings reinforce current gout management guidelines, which recommend continuation of urate-lowering therapy during acute flares and caution against unnecessary interruption [7]. The 2020 American College of Rheumatology (ACR) guideline specifically recommends starting ULT during a gout flare rather than waiting until it resolves, provided that anti-inflammatory treatment is given concurrently [7].

Despite clear guideline recommendations, deviations in inpatient practice remain common. Clinicians may discontinue allopurinol due to concerns about renal impairment or drug interactions, although dose adjustment rather than discontinuation is generally recommended [9-11]. Our findings suggest that such practices may contribute to preventable flares and associated morbidity.

Chronic kidney disease and diuretic use

In addition to medication-related factors, chronic kidney disease and diuretic use were independently associated with gout flares. These findings are biologically plausible, as impaired renal urate excretion and diuretic-induced hyperuricemia are well-established contributors to gout pathogenesis [1,10,11]. Diuretics, particularly loop and thiazide diuretics, reduce renal excretion of urate and have been associated with a two- to four-fold increased risk of incident or recurrent gout [12,13]. These associations are also supported by prior epidemiologic studies of diuretic exposure and gout risk [14-16]. The combination of CKD and diuretic use creates a particularly high-risk scenario for hospitalized patients with gout [4,5].

Serum uric acid levels

The elevated serum uric acid levels observed in patients who developed flares align with recent evidence demonstrating a graded relationship between serum urate concentration and recurrent gout flare risk [14]. Nearly all gout flares occur in patients with serum urate levels above 6 mg/dL, the therapeutic target recommended by major guidelines [7,14]. While serum uric acid was significantly elevated in univariate analysis, it did not remain an independent predictor in the multivariable model, likely due to its correlation with allopurinol management status and chronic kidney disease.

Clinical implications

Our findings have several important clinical implications: (a) Avoid unnecessary discontinuation of allopurinol in hospitalized patients with gout, as this substantially increases flare risk and contradicts current guideline recommendations; (b) Carefully manage initiation of therapy with concurrent anti-inflammatory prophylaxis (e.g., colchicine or low-dose non-steroidal anti-inflammatory drugs (NSAIDs)) when starting or resuming ULT during hospitalization [7,8]; (c) Recognize chronic kidney disease and diuretic use as high-risk factors requiring heightened vigilance for gout flares and consideration of prophylactic anti-inflammatory therapy; (d) Improve adherence to evidence-based guidelines in inpatient settings through education, clinical decision support systems, and standardized protocols for gout management; (e) Consider medication reconciliation at admission and discharge to ensure appropriate continuation of urate-lowering therapy.

Limitations

This study has several limitations. Its retrospective design limits causal inference and the ability to capture all relevant clinical variables. The single-center setting may affect generalizability to other hospital populations with different patient demographics and practice patterns. The sample size is relatively modest, and residual confounding cannot be excluded despite multivariable adjustment.

Additionally, detailed data on prophylactic anti-inflammatory therapy (colchicine, NSAIDs, or corticosteroids) were not systematically available, which may have influenced flare rates in patients with allopurinol modifications. The clinical definition of gout flare, while pragmatic for a retrospective study, may have led to some misclassification compared to validated flare definitions. Information on the presence of tophi, prior flare frequency, and duration of gout disease was not consistently available.

Future prospective studies with larger sample sizes, standardized protocols for gout management, and detailed capture of prophylactic therapy use are needed to confirm these findings and evaluate interventions to reduce inpatient gout flares.

## Conclusions

Gout flares are a common complication in hospitalized patients and are strongly associated with the modification of allopurinol therapy. Both discontinuation and initiation/resumption of allopurinol substantially increase flare risk, with approximately six- to seven-fold increased odds compared to continued therapy. Chronic kidney disease and diuretic use also independently predict flare risk.

Maintaining stable ULT and minimizing unnecessary interruptions, in accordance with current clinical practice guidelines from the ACR and European Alliance of Associations for Rheumatology (EULAR), may significantly reduce preventable inpatient gout flares and their associated morbidity. Healthcare systems should implement strategies to improve guideline-concordant care for hospitalized patients with gout, including education, clinical decision support, and standardized protocols that emphasize continuation of allopurinol therapy during hospitalization.
